# Automatic interictal epileptiform discharge (IED) detection based on convolutional neural network (CNN)

**DOI:** 10.3389/fmolb.2023.1146606

**Published:** 2023-04-07

**Authors:** Ling Zhang, Xiaolu Wang, Jun Jiang, Naian Xiao, Jiayang Guo, Kailong Zhuang, Ling Li, Houqiang Yu, Tong Wu, Ming Zheng, Duo Chen

**Affiliations:** ^1^ School of Innovation and Entrepreneurship, Hubei University of Science and Technology, Xianning, China; ^2^ School of Biomedical Engineering and Medical Imaging, Xianning Medical College, Hubei University of Science and Technology, Xianning, China; ^3^ Department of Clinical Neuroelectrophysiology, Wuhan Children’s Hospital of Tongji Medical College, Huazhong University of Science and Technology, Wuhan, China; ^4^ Department of Neurology, The Third Hospital of Xiamen, Xiamen, China; ^5^ Department of Neurology and Geriatrics, Fujian Institute of Geriatrics, Fujian Medical University Union Hospital, Fuzhou, China; ^6^ National Institute for Data Science in Health and Medicine, Xiamen University, Xiamen, China; ^7^ Department of Hematology, School of Medicine, Xiamen University, Xiamen, China; ^8^ School of Artificial Intelligence and Information Technology, Nanjing University of Chinese Medicine, Nanjing, China

**Keywords:** EEG, epilepsy, IED detection, deep learning, CNN

## Abstract

Clinical diagnosis of epilepsy significantly relies on identifying interictal epileptiform discharge (IED) in electroencephalogram (EEG). IED is generally interpreted manually, and the related process is very time-consuming. Meanwhile, the process is expert-biased, which can easily lead to missed diagnosis and misdiagnosis. In recent years, with the development of deep learning, related algorithms have been used in automatic EEG analysis, but there are still few attempts in IED detection. This study uses the currently most popular convolutional neural network (CNN) framework for EEG analysis for automatic IED detection. The research topic is transferred into a 4-labels classification problem. The algorithm is validated on the long-term EEG of 11 pediatric patients with epilepsy. The computational results confirm that the CNN-based model can obtain high classification accuracy, up to 87%. The study may provide a reference for the future application of deep learning in automatic IED detection.

## 1 Introduction

Epilepsy is a central nervous system (neurological) disorder in which brain activity becomes abnormal, causing seizures or periods of unusual behavior, sensations, and sometimes loss of awareness (Fisher et al., 2014). More than 70 million patients worldwide suffer from epilepsy accompany by about 2 million to 4 million new cases every year (Akyuz et al., 2021). Approximately 450,000 patients under the age of 17 are diagnosed with this disease out of nearly 3 million American patients (Galanopoulou et al., 2012). There are about 10 million epilepsy patients in China, and the first attack is mostly in children and adolescents (Ding et al., 2021).

The electroencephalogram (EEG) is a fundamental tool in the diagnosis and classification of epilepsy (Tatum et al., 2018). Epileptic brain activities include seizure and interictal epileptiform discharge (IED) (Horak et al., 2017). However, the availability of ictal EEGs is scarce for most seizures randomness, and uncertainty. Alternatively, IED is becoming one of the most important diagnostic hallmarks of epilepsy and can be used to localize epileptogenic foci, appearing mainly as spikes and sharp waves (de Curtis M et al., 2012). Half of the routine EEG recordings include IEDs, with this number even rising to 80% in sleep recordings from epilepsy patients (Westin et al., 2022).

IED is routinely assessed by visual analysis of the EEG by experts, considered the gold standard for many years. However, visual assessment is time-consuming and tends to be subjective, leading to misdiagnosis rates up to 30% (Lodder and van Putten, 2014) and motivating the development of computer-aided IED detection. An algorithm aimed to automatically detect IED started in 1976 based on scalp EEG. Extensive research on approaches to automatic IED detection has been carried out, ranging from mimetic methods to deep learning techniques (da Silva Lourenço et al., 2021).

Deep learning has been used in computer vision and speech recognition with automatic feature extraction and classification, which learn from the raw data without any *a priori* feature selection, scaling well to large datasets and exploiting hierarchical structure in natural signals (LeCun et al., 2015). Convolutional neural network (CNN) is the most widely-used deep learning method, which is increasingly popular in EEG analysis (Schirrmeister et al., 2017; Lawhern et al., 2018). There are some typical disadvantages of CNN, including false predictions output with high confidence, a large amount of training data, longer training time, a large number of hyperparameters (Thomas et al., 2021). In addition, the EEG signal is a dynamic and three-dimensional series in contrast to two-dimensional static images and has a comparatively low signal-to-noise ratio, which could make learning features in an end-to-end mode more difficult for EEG signals than for images (Lian and Xu, 2022). Despite the problems of CNN, the application of CNN still obtained some good results in EEG analysis. Several combinations of archetypes and variations of Convolutional and Recurrent Neural Networks also detect epileptiform discharges with high specificity (Tjepkema-Cloostermans et al., 2018). A VGG network shows high sensitivity and specificity in detecting epileptiform discharges, achieving intersections of metrics at 93% (Lourenço et al., 2020). The most representative work is the use of CNN in EEG decoding (Schirrmeister et al., 2017; Lawhern et al., 2018). The models in both reports achieved high classification accuracies on certain datasets.

Increasing studies attempt to use deep learning methods for EEG analysis. However, deep learning methods are still limitedly used in IED detection, a challenging but vital task in the diagnosis of epilepsy. Motivated by the good performance of the deep-learning-based models in EEG analysis, we here evaluate their effects on automatic IED detection. Several CNN-based frameworks are used to automatically annotate the IEDs from the long-term EEG recordings of 11 children with epilepsy. There are 3 types of IED in this study, i.e., spike and wave, spike, and low amplitude spike. With the non-IED EEG, the research in this paper can be transformed into a 4-labels classification problem. The computational results demonstrated an excellent classification accuracy of up to 87% on the validation set. This study may provide a reference for the future application of deep learning in automatic IED detection.

## 2 Materials and methods

### 2.1 Subjects

We retrospectively included 11 patients meeting the diagnosis standard in 2021 at Wuhan Children’s Hospital. All children with epilepsy also met the following inclusion criteria: 1) each patient with the video-EEG after treatment; 2) patients with 4 h of video-EEG monitoring, including a slow-wave sleeping period; 3) patients aged between 4 and 12 years old (mean ± std: 7 ± 3 years) during the video-EEG examination. The study was approved by the Research Ethics Board of Wuhan Children’s Hospital with IRB number 2022R034-E01.

### 2.2 Data acquisition

Using the standard international 10–20 system with 19 channels, EEG was recorded at a sampling rate of 200 Hz with a video-EEG system (Nihon Kohden). The non-IED, spike and wave, spike, and low amplitude spike are labeled as 0, 1, 2, and 3, respectively. All EEG clips were then shuffled into a random order, with all personal and identifying information completely removed.

### 2.3 Data preprocessing

**FIGURE 1:**
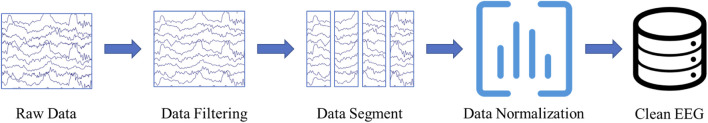
EEG Preprocessing. The raw EEG was band-pass filtered at [0.5, 50]*Hz*. A 1-s sliding window with 0.9-s overlap is used to crop the long-term EEG into segments.

Three experienced epileptologists manually read the long-term EEG and annotated the spike, low amplitude spike, spike and wave, and non-IED. A 64-order butter-worth band-pass filter ([0.5, 50]*Hz*) was enabled to eliminate the noises. The EEG was then cropped into segments using a 1-s sliding window with 0.9-s overlap, and then a z-score method was used to normalize the EEG data. Figure 1 illustrates the preprocessing. Table 1 summarizes the number and duration of IEDs of each subject after the preprocessing.

**TABLE 1:** Summary of subjects and IEDs.

Subject	Gender	Age (Year)	Number of IEDs	Duration summary (sec)
sub01	Male	10	135	192
sub02	Female	4	90	203
sub03	Male	11	124	149
sub04	Male	10	55	91
sub05	Female	5	131	163
sub06	Male	4	122	157
sub07	Female	12	87	473
sub08	Male	6	106	218
sub09	Male	10	114	302
sub10	Male	9	111	158
sub11	Male	12	131	268
Sum			1,206	2,373

### 2.4 Problem formulation

Since the EEG was manually annotated into four labels, the experimental task here was a 4-label classification problem. Given a multi-channel EEG dataset *X* with patient *i* ∈ {1, 2, … , *N*}, where *N* is the number of patients. Each dataset was divided into segments, as described earlier. Concretely, given dataset $D^{i}=\left(X^{1},y^{1}\right),\left(X^{2},y^{2}\right),\ldots ,\left(X^{N_{i}},y^{N_{i}}\right)$ , where *N*_*i*_ denotes the total number of segments for patient *i*. The *j*th EEG segment *X* ^*j*^ ∈ **R** ^*C*.*T*^, 1 ≤ *j* ≤ *N*_*i*_ contains *C* channels and *T* time points per segment, where *C* = 19 and *T* = 1 × 200 = 200, in this study. The class label of segment *j* is denoted by *y* ^*j*^ ∈ {0, 1, 2, 3}, corresponding to non-IED, spike and wave, spike, and low amplitude spike.

The EEG segments were divided under a 10-fold cross-validation strategy. The dataset was divided into ten portions. In each repeated iteration, one portion of the data was randomly used as testing data and the rest nine portions of the data was applied as training data. This process would be repeated 10 times until all data had been tested once. The classification performance was evaluated by aggregating all iterations. The approach was carried out with Tensorflow 2.10.0. For training models, Adam was used with a batch of 10,000 EEG segments and 500 epochs. The drop-out rate was 0.5 for the protocol.

### 2.5 Convolutional neural network

**FIGURE 2:**
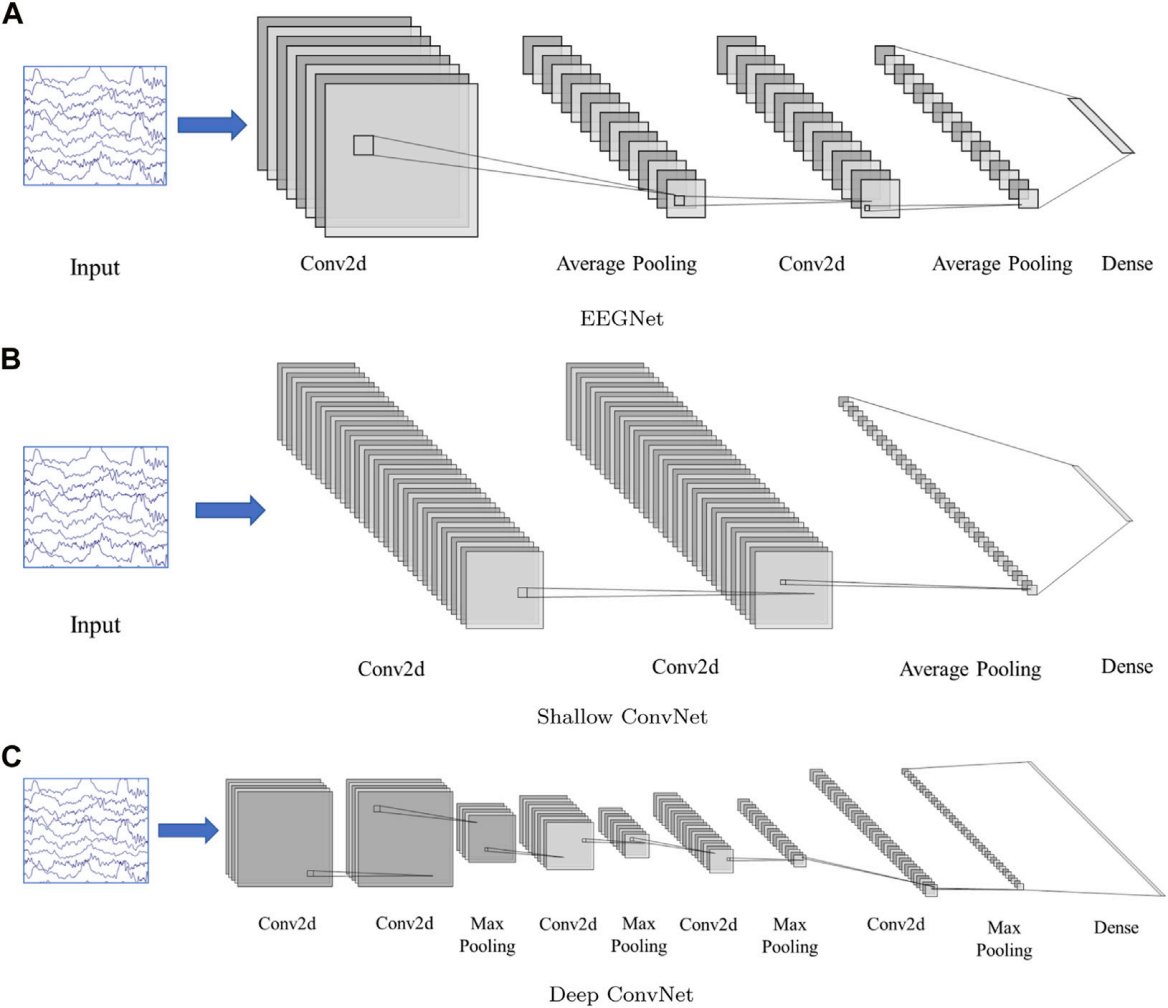
Network structure. **(A)**: EEGNet, **(B)**: Shallow ConvNet, **(C)**: Deep ConvNet. The input is multi-channel EEG segment with a dimension 19(channel) × 200(sampling point, 1 × 200). The EEGNet and Shallow ConvNet both contain two 2D convolution layers, while Deep ConvNet contains 5. The structure details can be found in (Schirrmeister et al., 2017; Lawhern et al., 2018)

Three CNN-based frameworks are used in this study, including, EEGNet (Lawhern et al., 2018), Deep ConvNet, and Shallow ConvNet (Schirrmeister et al., 2017). The model structure can be found in Figure 2.

All models were trained on an NVIDIA A40 GPU, with CUDA 11.8 and cuDNN v8, in tensorflow-gpu (v 2.10.0). Bias units are left out in all convolutional layers. Two-dimensional convolution functions are used for easy software implementation.

### 2.6 Model evaluation

In this paper, the EEG clips have four classes, i.e., non-IED, spike and wave, spike, and low amplitude spike, which were labeled as 0, 1, 2, and 3, respectively. A confusion matrix and three metrics (accuracy, precision, and recall) are used to evaluate the algorithm performance. A confusion matrix was used to visualize the overall classification results. Accuracy was used to count the probability of the samples being correctly identified in the EEG clips. In addition, precision, and recall were used to further evaluate the algorithm performance in each class.

For example, if the class “spike” was considered as “positive,” all the other samples would be considered as “negative”. Therefore, the classifier has 4 possible outcomes: True positive (TP), false positive (FP), true negative (TN), and false negative (FN). The accuracy, precision, and recall were calculated as follows:

$$\begin{array}{ll} & Precision=\frac{TP}{TP+FP}\\ & Recall=\frac{TP}{TP+FN} \end{array}$$ (1)

In a similar way, we can calculate the precision and recall for the other three classes.

## 3 Results and discussion

### 3.1 Performance evaluation

A total of 11 patients (8 boys and 3 girls) with epilepsy were finally included (age range: 4–12 years, mean age, 7 years, standard deviation: 3 years). A total of 11 4-h video-EEG datasets were obtained. The classification performance of the three models was tested on both the validation set and training set. Table 2 displays the result for evaluating the score on the two sets, indicating a relatively good detection effect with the three models. The Shallow ConvNet achieved the highest mean accuracy in evaluating the score automatically both on the validation set with a 10-fold cross-validation strategy and the training set. The accuracy is 87.0% and 84.8%, respectively. Recall and precision generally reflected the proportion of true positive, which also shows a similar superior performance on the Shallow ConvNet. It is worth noting that the models carried out a suitable trade-off in four-class scores.

**TABLE 2:** The performance on training and validation datasets.

Type	Dataset	Accuracy (%)	Recall (%)	Precision (%)
EEGNet	Training set	78.2	72.3	81.9
	Validate set	80.5%	74.7%	83.9%
Shallow ConvNet	Training set	84.8%	86.3%	85.6%
	Validate set	87.0%	86.2%	87.6%
Deep ConvNet	Training set	83.5%	82.8%	84.1%
	Validate set	70.6%	69.1%	72.5%

### 3.2 Multi labels classification performance evaluation

**FIGURE 3:**
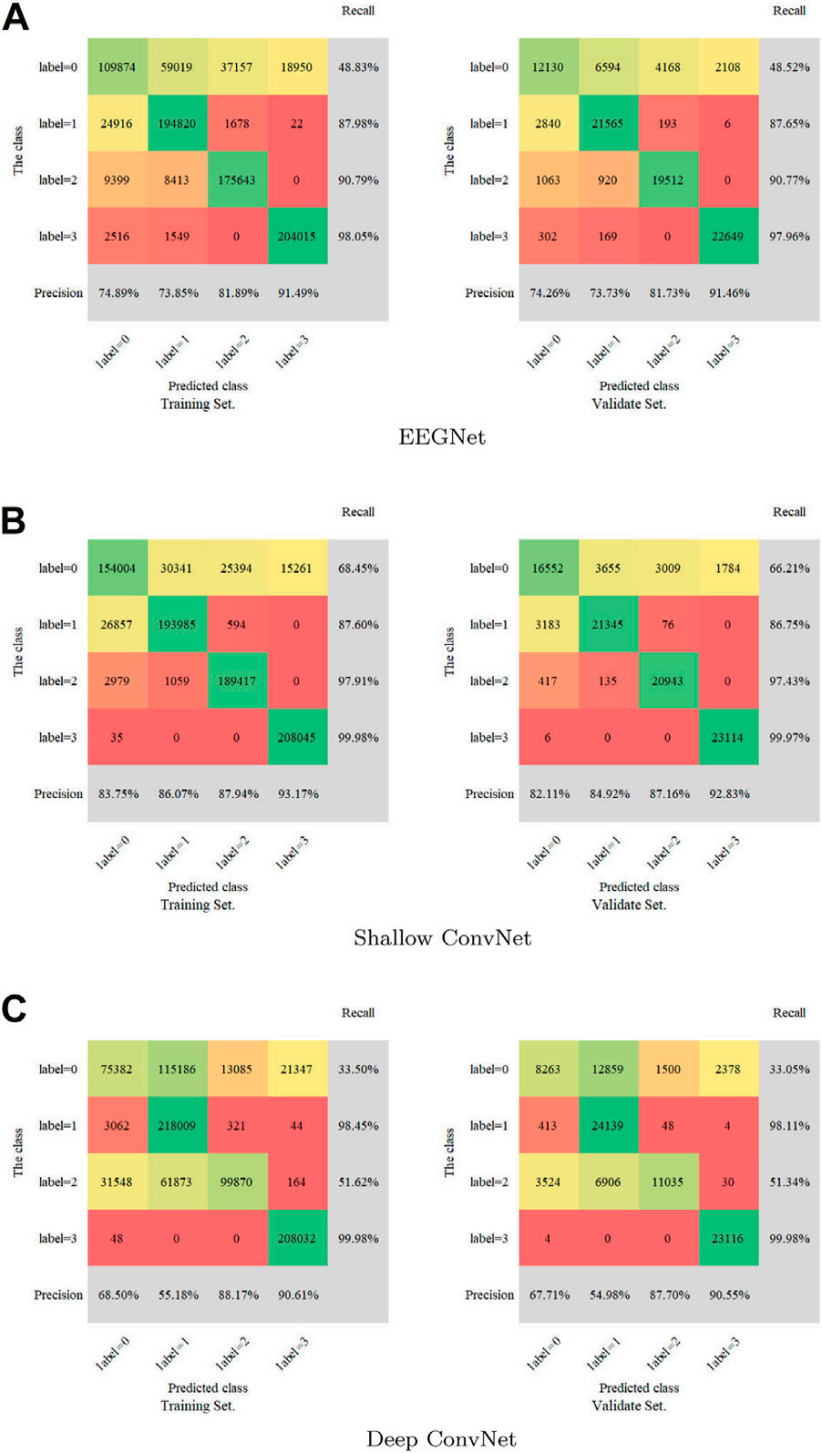
Confusion matrix for the training set and validation set. **(A)**: EEGNet, **(B)**: Shallow ConvNet, **(C)**: Deep ConvNet. Labels 0-4 represent non-IED, spike and wave, spike, and low amplitude spike, respectively.

A confusion matrix as a metric was introduced to measure the multi-label classification performance of the three models. The full confusion matrix of the training set and validation set are shown in Figure 3, including precision and recall for quantification. Some misclassifications are presented. Among all the attempts, the highest recall rate is 99.98%, while the lowest rate is 33.05%. The maximum was 93.17% and the minimum was 54.98% for precision.

**FIGURE 4:**
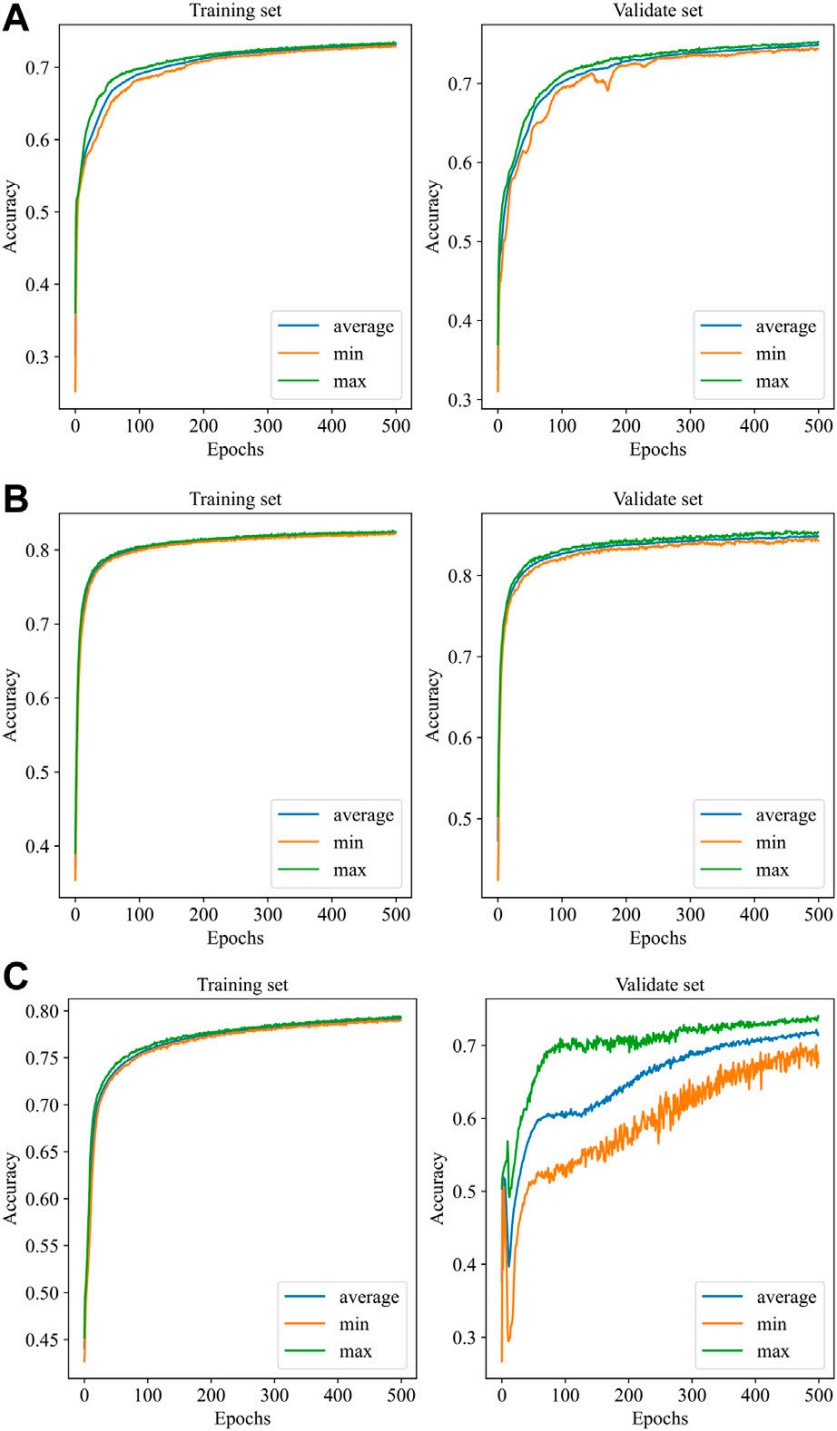
Performance of the models with 10-fold cross-validation. **(A)**: EEGNet, **(B)**: Shallow ConvNet, **(C)**: Deep ConvNet. In each subfigure, the blue curve is the average of the 10 subfolders, while the green and the orange curves represent the ± 1 standard deviation.

Figure 4 illustrates the performance of the three models in the 10-fold cross-validation. As described, the experiment was repeated 10× to obtain the averaged results. It is obvious that the accuracy of each test was much higher than the chance level (1/4 = 25%) in the validation set. The highest and lowest accuracy showed 87.0% and 70.6%, while the highest accuracy was 84.8% accompanied by 78.2% lowest accuracy in the training set.

### 3.3 Method comparison

To our best knowledge, there are few deep learning methods for automated EEG analysis of IED in epilepsy diagnostics. In this study, we use the CNN framework for automatic IED detection from EEG data. To validate the effectiveness of the proposed framework, we use Shallow ConvNet for automatic IED detection. As a comparison method, we also implement deep neural network (Tjepkema-Cloostermans et al., 2018), which used several combinations of convolutional and recurrent neural networks to detect epileptiform discharges from scalp EEG data. For readability sake, we select the best models in the two research for comparison, i.e., Shallow ConvNet in our study, and Deep Neural Network in (Tjepkema-Cloostermans et al., 2018). The classification performance of the two models was tested on both the validation set and the training set. Table 3 displays the performance comparison of Shallow ConvNet and Deep Neural Network. The proposed Shallow ConvNet achieves an 87% accuracy on validate set with a 10-fold cross-validation strategy, while the compared method Deep Neural Network has an accuracy of 77.02%. This demonstrates the effectiveness of Shallow ConvNet in detecting IED on EEG data.

**TABLE 3:** Method comparison on training and validation datasets.

Method	Dataset	Accuracy (%)	Recall (%)	Precision (%)
Deep Neural Network	Training set	79.89	79.89	79.89
	Validate set	77.02	77.16	76.86
Shallow ConvNet	Training set	84.80	86.30	85.60
	Validate set	87.00	86.20	87.60

### 3.4 Discussion

Automatic IED detection based on CNN is gaining attention for clinical auxiliary diagnosis of epilepsy in the future. Here, three CNN-based models are evaluated for IED detection. The scalp EEG was data as the input of the CNN detector. The performance of Shallow ConvNet was obviously best good and considered to be promising by the accuracy of over 84.0%. Precision and/or recall was consistent at over 85.0%, being very promising. The accuracy and precision of EEGNet performed well at about 80%, while recall was relatively low at about 73.5%. The Deep ConvNet showed good performance (83.5%, 82.8%, 84.1%) in the training set while fluctuating substantially in the validation set (70.6%, 69.1%, 72.5%), indicating running more epochs to converge (Figure 4).

All EEG segments were classified according to spike, low amplitude spike, spike and wave, and non-IED. It is friendly to label 3 with over 97% recall and over 90% precision. The label 0 is more likely to be misclassified with about 33% recall in the Deep ConvNet, 48% in the EEGNet, and 67% in the Shallow ConvNet. Precision is higher than 82% for four classifications in the Shallow ConvNet. Although precision is only about 68% and 55% for labels 0 and 1 in the Deep ConvNet, about 68% and 55% for labels 0 and 1 in the EEGNet. It is good for labels 3 and 4 with a consistent precision of over 81% and 91%, indicating different advantages of the three models. The three models used here perform well in IED identification. The results verified that the Shallow ConvNet model showed outstanding performance for the 4-label classification problem, displaying its good stability, in the IED detection here. The EEGNet and Deep ConvNet performed well in limited classification, indicating promising CNN-based automatic IED detection.

Apparently, there are still some limitations in our study. The raw EEG data here only came from 11 children with epilepsy. It requires proving whether the models can be applied to all the EEG datasets of all patients with epilepsy. The sample size was still small in contrast to large-sample clinical trials for clinical application. Further optimization must be considered for its computational cost.

## 4 Conclusion

Rapid progress in neuroimaging techniques and deep learning with CNN has significantly enhanced research on automatic IED detection. Our study investigated the usability of three different CNN-based models in a 4-label classification problem for automatic IED detection. A remarkable classification accuracy of above 87% was achieved for the Shallow ConvNet. The EEGNet and Deep ConvNet also showed advantages in certain classes. Further research will focus on the interpretability of the layers and the optimization of the model structure.

## Data Availability

The raw data supporting the conclusion of this article will be made available by the authors, without undue reservation.
